# Supplementary material for: Developmental Biology and Identification of a Garden Pest, Otiorhynchus (Podoropelmus) smreczynskii Cmoluch, 1968 (Coleoptera, Curculionidae, Entiminae), with Comments on Its Origin and Distribution
Source: Insects. 2023 Apr 4;14(4):360. doi: 10.3390/insects14040360 (PMC10147090; doi:10.3390/insects14040360)
Supplement: Supplementary file 1 [file insects-14-00360-s001.zip › Supplement file 1.pdf]

Supplementary file S1.
List of *Otiorhynchus* specimens used for molecular study sorted by voucher code, species name, locality, host plant affiliation, *COI* haplotype name with GenBank accession number; and haplotype frequency.

voucher code	Species	Locality	Host plant	Haplotype name (NCBI accession number)	haplotype frequency
4633	*Otiorhynchus smreczynskii* Cmol.	Poland, Skrzynice, N51.12245, E22.67538, 6.08.2017, lgt. R. Gosik	*Ligustrum vulgare* L.	**smr1 (OQ382848)**	**55**
4634	*Otiorhynchus smreczynskii* Cmol.	Germany, Hannover, 30.06.2017, lgt. Stein Tormasch	*Lonicera pileata* Oliv.		
4635	*Otiorhynchus smreczynskii* Cmol.	Germany, Hannover, 30.07.2017, lgt. P. Sprick	*Lonicera pileata* Oliv.		
4636	*Otiorhynchus smreczynskii* Cmol.	Germany, Hannover, 30.07.2017, lgt. P. Sprick	*Lonicera pileata* Oliv.		
4637	*Otiorhynchus smreczynskii* Cmol.	Poland, Lublin, Botanic Garden, N51.26432, E22.51586, 29.07.2017, lgt. R. Gosik	*Ligustrum vulgare* L.		
4638	*Otiorhynchus smreczynskii* Cmol.	Poland, Lublin, Botanic Garden, N51.26432, E22.51586, 29.07.2017, lgt. R. Gosik	*Syringa vulgaris* L.		
4639	*Otiorhynchus smreczynskii* Cmol.	Poland, Lublin, Botanic Garden, N51.26432, E22.51586, 29.07.2017, lgt. R. Gosik	*Syringa vulgaris* L.		
4640	*Otiorhynchus smreczynskii* Cmol.	Poland, Lublin, Botanic Garden, N51.26432, E22.51586, 29.07.2017, lgt. R. Gosik	*Syringa vulgaris* L.		
4641	*Otiorhynchus smreczynskii* Cmol.	Poland, Lublin, Botanic Garden, N51.26432, E22.51586, 29.07.2017, lgt. R. Gosik	*Syringa vulgaris* L.		
6419	*Otiorhynchus smreczynskii* Cmol.	Czechia, Bohemia or., Hradec Králove, Náměstí 5 května, N50 12.447 E15 49.384, 30.08.2022 lgt. J. Krátký	*Ligustrum vulgare* L.		
6420	*Otiorhynchus smreczynskii* Cmol.	Czechia, Bohemia or., Hradec Králove, Náměstí 5 května, N50 12.447 E15 49.384, 30.08.2022 lgt. J. Krátký	*Ligustrum vulgare* L.		
6421	*Otiorhynchus smreczynskii* Cmol.	Czechia, Bohemia or., Hradec Králove, Náměstí 5 května, N50 12.447 E15 49.384, 30.08.2022 lgt. J. Krátký	*Ligustrum vulgare* L.		
6422	*Otiorhynchus smreczynskii* Cmol.	Czechia, Bohemia or., Hradec Králove, Náměstí 5 května, N50 12.447 E15 49.384, 30.08.2022 lgt. J. Krátký	*Ligustrum vulgare* L.		
6426	*Otiorhynchus smreczynskii* Cmol.	Germany, Eberswalde, N52.844157 E13.806817, 4.08.2022, lgt. Jakob Jilg.	*Lonicera pileata* Oliv.		
6427	*Otiorhynchus smreczynskii* Cmol.	Germany, Eberswalde, N52.844157 E13.806817, 4.08.2022, lgt. Jakob Jilg.	*Lonicera pileata* Oliv.		
6428	*Otiorhynchus smreczynskii* Cmol.	Czechia, Bohemia C., Kostelec nad Labem, Havlíčkova 1117, 11.06.2022, P. Jansa lgt., Oklep	*Ligustrum vulgare* L.		
6429	*Otiorhynchus smreczynskii* Cmol.	Czechia, Bohemia C., Kostelec nad Labem, Havlíčkova 1117, 11.06.2022, P. Jansa lgt., Oklep	*Ligustrum vulgare* L.		
6430	*Otiorhynchus smreczynskii* Cmol.	Czechia, Bohemia C., Kostelec nad Labem, Havlíčkova 1117, 11.06.2022, P. Jansa lgt., Oklep	*Ligustrum vulgare* L.		

6431	*Otiorhynchus smreczynskii* Cmol.	Czechia, Bohemia C., Kostelec nad Labem, Havličkova 1117, 11.06.2022, P. Jansa lgt., Oklep	*Ligustrum vulgare* L.		
6432	*Otiorhynchus smreczynskii* Cmol.	Czechia, Bohemia C., Kostelec nad Labem, Havličkova 1117, 11.06.2022, P. Jansa lgt., Oklep	*Ligustrum vulgare* L.		
6433	*Otiorhynchus smreczynskii* Cmol.	Czechia, Bohemia C., Kostelec nad Labem, Havličkova 1117, 11.06.2022, P. Jansa lgt., Oklep	*Ligustrum vulgare* L.		
6434	*Otiorhynchus smreczynskii* Cmol.	Czechia, Bohemia c. 5752, Libiš - okoli čarpaci stanice na Pražské ulici 30.05.2022, P. Jansa lgt., Oklep	*Ligustrum vulgare* L.		
6435	*Otiorhynchus smreczynskii* Cmol.	Czechia, Bohemia c. 5752, Libiš - okoli čarpaci stanice na Pražské ulici 30.05.2022, P. Jansa lgt., Oklep	*Ligustrum vulgare* L.		
6436	*Otiorhynchus smreczynskii* Cmol.	Czechia, Bohemia c. 5752, Libiš - okoli čarpaci stanice na Pražské ulici 30.05.2022, P. Jansa lgt., Oklep	*Ligustrum vulgare* L.		
6437	*Otiorhynchus smreczynskii* Cmol.	Czechia, Bohemia c. 5752, Libiš - okoli čarpaci stanice na Pražské ulici 30.05.2022, P. Jansa lgt., Oklep	*Ligustrum vulgare* L.		
6438	*Otiorhynchus smreczynskii* Cmol.	Germany, Hannover bug., Le de buvg. 9.07.2022, lgt. P. Sprick	*Ligustrum ovalifolium* Hassk		
6439	*Otiorhynchus smreczynskii* Cmol.	Germany, Neu bleyen (Klistrain), 13.08.2022, lgt. P. Sprick	*Ligustrum ovalifolium* Hassk		
6440	*Otiorhynchus smreczynskii* Cmol.	Germany, Hannover, Steintosmasch, 9.07.2022, lgt. P. Sprick	*Ligustrum ovalifolium* Hassk		
6441	*Otiorhynchus smreczynskii* Cmol.	Germany, Kirstil Keitz, Rosch strg 3c, 13.08.2022, lgt. P. Sprick	*Syringa vulgaris* L.		
6442	*Otiorhynchus smreczynskii* Cmol.	Germany, Neubleyen, Cklistrin kelb. 13.08.2022, lgt. P. Sprick	*Syringa vulgaris* L.		
6443	*Otiorhynchus smreczynskii* Cmol.	Germany, Klistrin-Kietz, W, Bahnhof, 13.08.2022, lgt. P. Sprick	*Syringa vulgaris* L.		
6444	*Otiorhynchus smreczynskii* Cmol.	Germany, Hannover-Burg, Lederburg, 9.07.2022, lgt. P. Sprick	*Syringa vulgaris* L.		
6445	*Otiorhynchus smreczynskii* Cmol.	Germany, Klistrin-Kietz, Wilhelm strasse 3c, 13.08.2022 lgt. P. Sprick	*Syringa vulgaris* L.		
6446	*Otiorhynchus smreczynskii* Cmol.	Poland, Lublin, Dunikowskiego, N51.21839, E22.57170, 24.05.2022, lgt. R. Gosik	*Ligustrum vulgare* L.		
6447	*Otiorhynchus smreczynskii* Cmol.	Poland, Lublin, Dunikowskiego, N51.21839, E22.57170, 24.05.2022, lgt. R. Gosik	*Ligustrum vulgare* L.		
6448	*Otiorhynchus smreczynskii* Cmol.	Poland, Lublin, Dunikowskiego, N51.21839, E22.57170, 24.05.2022, lgt. R. Gosik	*Ligustrum vulgare* L.		
6449	*Otiorhynchus smreczynskii* Cmol.	Poland, Lublin, Dunikowskiego, N51.21839, E22.57170, 24.05.2022, lgt. R. Gosik	*Ligustrum vulgare* L.		
6450	*Otiorhynchus smreczynskii* Cmol.	Poland, Lublin, Dunikowskiego, N51.21839, E22.57170, 24.05.2022, lgt. R. Gosik	*Ligustrum vulgare* L.		
6451	*Otiorhynchus smreczynskii* Cmol.	Poland, Lublin, Dunikowskiego, N51.21839E, E22.57170, 24.05.2022, lgt. R. Gosik	*Ligustrum vulgare* L.		
6452	*Otiorhynchus smreczynskii* Cmol.	Poland, Warszawa, Połczyńska, N52.22142, E20.91870, 9.06.2022, lgt. R. Gosik	*Ligustrum vulgare* L.		

6453	***Otiorhynchus smreczynskii* Cmol.**	**Poland, Warszawa, Połczyńska, N52.22142, E20.91870, 9.06.2022, lgt. R. Gosik**	***Ligustrum vulgare* L.**		
6454	***Otiorhynchus smreczynskii* Cmol.**	**Poland, Warszawa, Połczyńska, N52.22142, E20.91870, 9.06.2022, lgt. R. Gosik**	***Ligustrum vulgare* L.**		
6455	***Otiorhynchus smreczynskii* Cmol.**	**Poland, Warszawa, Połczyńska, N52.22142, E20.91870, 9.06.2022, lgt. R. Gosik**	***Ligustrum vulgare* L.**		
6456	***Otiorhynchus smreczynskii* Cmol.**	**Poland, Warszawa, Połczyńska, N52.22142, E20.91870, 9.06.2022, lgt. R. Gosik**	***Ligustrum vulgare* L.**		
6457	***Otiorhynchus smreczynskii* Cmol.**	**Poland, Warszawa, Połczyńska, N52.22142, E20.91870, 9.06.2022, lgt. R. Gosik**	***Ligustrum vulgare* L.**		
6458	***Otiorhynchus smreczynskii* Cmol.**	**Poland, Kraków, Szlak, N50.07145, E19.93854, 23.06.2022, lgt. R. Gosik**	***Ligustrum vulgare* L.**		
6459	***Otiorhynchus smreczynskii* Cmol.**	**Poland, Kraków, Szlak, N50.07145, E19.93854, 23.06.2022, lgt. R. Gosik**	***Ligustrum vulgare* L.**		
6460	***Otiorhynchus smreczynskii* Cmol.**	**Poland, Kraków, Szlak, N50.07145, E19.93854, 23.06.2022, lgt. R. Gosik**	***Ligustrum vulgare* L.**		
6461	***Otiorhynchus smreczynskii* Cmol.**	**Poland, Kraków, Szlak, N50.07145, E19.93854, 23.06.2022, lgt. R. Gosik**	***Ligustrum vulgare* L.**		
6462	***Otiorhynchus smreczynskii* Cmol.**	**Poland, Kraków, Szlak, N50.07145, E19.93854, 23.06.2022, lgt. R. Gosik**	***Ligustrum vulgare* L.**		
6463	***Otiorhynchus smreczynskii* Cmol.**	**Poland, Kraków, Szlak, N50.07145, E19.93854, 23.06.2022, lgt. R. Gosik**	***Ligustrum vulgare* L.**		
6470	***Otiorhynchus smreczynskii* Cmol.**	**Poland, Rucione-Nida, N53.63993, E21.54440, 2.07.2022, lgt. R. Gosik**	***Ligustrum vulgare* L.**		
6471	***Otiorhynchus smreczynskii* Cmol.**	**Poland, Kraków, Prądnik, N50.09283, E19.97604, 23.06.2022, lgt. R. Gosik**	***Syringa vulgaris* L.**		
6472	***Otiorhynchus smreczynskii* Cmol.**	**Poland, Kraków, Prądnik, N50.09283, E19.97604, 23.06.2022, lgt. R. Gosik**	***Syringa vulgaris* L.**		
6473	***Otiorhynchus smreczynskii* Cmol.**	**Poland, Kraków, Prądnik, N50.09283, E19.97604, 23.06.2022, lgt. R. Gosik**	***Syringa vulgaris* L.**		
6464	***Otiorhynchus rotundus* Marsh.**	**Poland, Rucione-Nida, N53.63993, E21.54440, 2.07.2022, lgt. R. Gosik**	***Ligustrum vulgare* L.**	**rot1 (OQ382849)**	**6**
6465	***Otiorhynchus rotundus* Marsh.**	**Poland, Rucione-Nida, N53.63993, E21.54440, 2.07.2022, lgt. R. Gosik**	***Ligustrum vulgare* L.**		
6466	***Otiorhynchus rotundus* Marsh.**	**Poland, Rucione-Nida, N53.63993, E21.54440, 2.07.2022, lgt. R. Gosik**	***Ligustrum vulgare* L.**		
6467	***Otiorhynchus rotundus* Marsh.**	**Poland, Rucione-Nida, N53.63993, E21.54440, 2.07.2022, lgt. R. Gosik**	***Ligustrum vulgare* L.**		
6468	***Otiorhynchus rotundus* Marsh.**	**Poland, Rucione-Nida, N53.63993, E21.54440, 2.07.2022, lgt. R. Gosik**	***Ligustrum vulgare* L.**		
6469	***Otiorhynchus rotundus* Marsh.**	**Poland, Rucione-Nida, N53.63993, E21.54440, 2.07.2022, lgt. R. Gosik**	***Ligustrum vulgare* L.**		
6474	***Otiorhynchus rotundus* Marsh.**	**Poland, Gródek, N50.80711, E23.95661, 27.05.2022, lgt. R. Gosik**	***Syringa vulgaris* L.**	**rot2 (OQ382850)**	**6**
6475	***Otiorhynchus rotundus* Marsh.**	**Poland, Gródek, N50.80711, E23.95661, 27.05.2022, lgt. R. Gosik**	***Syringa vulgaris* L.**		
6476	***Otiorhynchus rotundus* Marsh.**	**Poland, Gródek, N50.80711, E23.95661, 27.05.2022, lgt. R. Gosik**	***Syringa vulgaris* L.**		
6477	***Otiorhynchus rotundus* Marsh.**	**Poland, Gródek, N50.80711, E23.95661, 27.05.2022, lgt. R. Gosik**	***Syringa vulgaris* L.**		
6478	***Otiorhynchus***	**Poland, Gródek, N50.80711,**	***Syringa vulgaris* L.**		

	rotundus Marsh.	E23.95661, 27.05.2022, lgt. R. Gosik			
6479	*Otiorhynchus rotundus* Marsh.	Poland, Gródek, N50.80711, E23.95661, 27.05.2022, lgt. R. Gosik	*Syringa vulgaris* L.		
