# Supplementary material for: Developmental Biology and Identification of a Garden Pest, Otiorhynchus (Podoropelmus) smreczynskii Cmoluch, 1968 (Coleoptera, Curculionidae, Entiminae), with Comments on Its Origin and Distribution
Source: Insects. 2023 Apr 4;14(4):360. doi: 10.3390/insects14040360 (PMC10147090; doi:10.3390/insects14040360)
Supplement: Supplementary file 1 [file insects-14-00360-s001.zip › Supplement file 2.pdf]

Supplementary file S2.

Detailed results of measurements of adults of *O. smreczynskii* (48 exx.) and *O. rotundus* (30 exx.)

Species	Deposit.	sex	Locality	latitude	longitude	Date	leg./det.	abdomen length (A)	abdomen width (B)	thorax length (C)	thorax width (D)	head and rostrum length (E)	head width (F)	first tibia length (G)	funiculus and clava (H)	length of the body (I)	A/B	C/D
O. smreczynskii Cmol.	UMCS	f	holotyp	N51.2610	E22.5150	16.09.1965	Z. Cmoluch	3,35	2,80	1,40	1,70	1,25	0,95	1,50	1,60	5,40	1,1964285	0,8235294
O. smreczynskii Cmol.	UMCS	f	Poland, Lublin	N51.2610	E22.5150	11.06.1973	Z. Cmoluch	3,50	2,95	1,65	1,75	1,25	1,00	1,55	2,00	5,10	1,186440678	0,942857143
O. smreczynskii Cmol.	UMCS	f	Poland, Lublin	N51.2610	E22.5150	15.07.1976	Z. Cmoluch	3,00	2,50	1,35	1,45	1,20	0,90	1,35	1,55	5,00	1,2	0,931034483
O. smreczynskii Cmol.	UMCS	f	Poland, Lublin	N51.2610	E22.5150	15.07.1976	Z. Cmoluch	3,05	2,50	1,45	1,50	1,15	0,85	1,30	1,60	5,10	1,22	0,966666667
O. smreczynskii Cmol.	UMCS	f	Poland, Lublin	N51.2610	E22.5150	15.07.1976	Z. Cmoluch	3,00	2,50	1,20	1,45	1,20	0,85	1,25	1,50	5,50	1,2	0,827586207
O. smreczynskii Cmol.	UMCS	f	Poland, Lublin	N51.2610	E22.5150	15.07.1976	Z. Cmoluch	3,20	2,50	1,30	1,45	1,20	0,85	1,45	1,70	5,00	1,28	0,896551724
O. smreczynskii Cmol.	UMCS	f	Poland, Lublin	N51.2610	E22.5150	15.07.1976	Z. Cmoluch	2,90	2,25	1,30	1,40	1,10	0,85	1,30	1,50	4,50	1,288888889	0,928571429
O. smreczynskii Cmol.	UMCS	f	Poland, Lublin	N51.2610	E22.5150	15.07.1976	Z. Cmoluch	3,05	2,50	1,35	1,40	1,15	0,80	1,35	1,80	4,90	1,22	0,964285714
O. smreczynskii Cmol.	UMCS	f	Poland, Lublin	N51.2610	E22.5150	15.07.1976	Z. Cmoluch	3,00	2,70	1,40	1,55	1,15	0,90	1,45	1,50	5.00	1,111111111	0,903225806
O. smreczynskii Cmol.	UMCS	f	Poland, Lublin	N51.2610	E22.5150	15.07.1976	Z. Cmoluch	3,05	2,50	1,40	1,55	1,25	0,90	1,35	1,70	5.00	1,22	0,903225806
O. smreczynskii Cmol.	UMCS	f	Poland, Lublin	N51.2610	E22.5150	17.07.1979	Z. Cmoluch	3,00	2,40	1,25	1,35	1,10	0,80	1,45	1,50	4,90	1,25	0,925925926
O. smreczynskii Cmol.	UMCS	f	Poland, Lublin	N51.2610	E22.5150	17.07.1979	Z. Cmoluch	2,80	2,40	1,25	1,25	1,00	0,85	1,35	1,50	5,00	1,166666667	1
O. smreczynskii Cmol.	UMCS	f	Poland, Lublin	N51.2610	E22.5150	17.07.1979	Z. Cmoluch	2,75	2,35	1,25	1,30	1,05	0,75	1,00	1,30	4,90	1,170212766	0,961538462
O. smreczynskii Cmol.	UMCS	f	Poland, Lublin	N51.2610	E22.5150	17.07.1979	Z. Cmoluch	3,15	2,40	1,35	1,50	1,10	0,85	1,40	1,35	5,00	1,3125	0,9
O. smreczynskii Cmol.	UMCS	f	Poland, Lublin	N51.2610	E22.5150	17.07.1979	Z. Cmoluch	3,00	2,50	1,40	1,50	1,15	0,90	1,35	1,50	5,10	1,2	0,933333333
O. smreczynskii Cmol.	UMCS	f	Poland, Lublin	N51.2610	E22.5150	17.07.1979	Z. Cmoluch	2,75	2,40	1,25	1,45	1,20	0,90	1,35	1,50	4,90	1,145833333	0,862068966
O. smreczynskii Cmol.	UMCS	f	Poland, Lublin	N51.2610	E22.5150	17.07.1979	Z. Cmoluch	3,15	2,50	1,35	1,45	1,25	0,85	1,45	1,50	5,00	1,26	0,931034483
O. smreczynskii Cmol.	UMCS	f	Poland, Lublin	N51.2610	E22.5150	17.07.1979	Z. Cmoluch	3,00	2,55	1,35	1,50	1,25	0,95	1,30	1,50	5,20	1,176470588	0,9
O. smreczynskii Cmol.	UMCS	f	Poland, Lublin	N51.2610	E22.5150	17.07.1979	Z. Cmoluch	3,25	2,50	1,40	1,50	1,05	0,85	1,40	1,60	5,10	1,3	0,933333333
O. smreczynskii Cmol.	UMCS	f	Poland, Lublin	N51.2610	E22.5150	17.07.1979	Z. Cmoluch	2,65	2,25	1,15	1,30	1,10	0,80	1,25	1,60	4,40	1,177777778	0,884615385
O. smreczynskii Cmol.	UMCS	f	Poland, Lublin	N51.2610	E22.5150	17.07.1979	Z. Cmoluch	3,75	2,70	1,50	1,65	1,20	0,90	1,50	1,75	5,40	1,388888889	0,909090909
O. smreczynskii Cmol.	UMCS	f	Poland, Lublin	N51.2610	E22.5150	17.07.1979	Z. Cmoluch	3,25	2,50	1,45	1,50	1,10	0,85	1,50	1,55	5,10	1,3	0,966666667
O. smreczynskii Cmol.	UMCS	f	Poland, Lublin	N51.2610	E22.5150	17.07.1979	Z. Cmoluch	3,25	2,55	1,55	1,50	1,25	0,90	1,25	1,50	5,20	1,274509804	1,033333333
O. smreczynskii Cmol.	UMCS	f	Poland, Lublin	N51.2610	E22.5150	17.07.1979	Z. Cmoluch	2,90	2,40	1,40	1,40	1,25	0,90	1,35	1,50	4,70	1,208333333	1

O. smreczynskii Cmol.	UMCS	f	Poland, Lublin	N51.2610E	E22.5150	17.07.1979	Z. Cmoluch	3,25	2,50	1,35	1,40	1,05	0,85	1,45	1,50	5,10	1,3	0,964285714
O. smreczynskii Cmol.	UMCS	f	Poland, Lublin	N51.2610E	E22.5150	17.07.1979	Z. Cmoluch	3,40	2,85	1,50	1,60	1,05	0,90	1,50	1,70	5,30	1,192982456	0,9375
O. smreczynskii Cmol.	UMCS	f	Poland, Lublin	N51.2610	E22.5150	17.07.1979	Z. Cmoluch	3,10	2,55	1,35	1,50	1,05	0,90	1,45	1,70	5,00	1,215686275	0,9
O. smreczynskii Cmol.	UMCS	f	Poland, Lublin	N51.2610	E22.5150	17.07.1979	Z. Cmoluch	3,25	2,55	1,45	1,55	1,20	0,95	1,50	1,55	5,00	1,274509804	0,935483871
O. smreczynskii Cmol.	UMCS	f	Poland, Lublin	N51.2610	E22.5150	17.07.1979	Z. Cmoluch	3,00	2,50	1,50	1,55	1,25	0,95	1,50	1,60	4,90	1,2	0,967741935
O. smreczynskii Cmol.	UMCS	f	Poland, Lublin	N51.2430	E22.540432	10.06.1983	B. Staniec	3,50	2,85	1,60	1,65	1,05	1,00	1,55	1,85	5,30	1,228070175	0,96969697
O. smreczynskii Cmol.	UMCS	f	Poland, Lublin	N51.2118	E22.5715	10.06.2021	R. Gosik	2,95	2,40	1,40	1,45	1,25	0,85	1,35	1,60	4,90	1,229166667	0,965517241
O. smreczynskii Cmol.	UMCS	f	Poland, Lublin	N51.2118	E22.5715	10.06.2021	R. Gosik	3,25	2,70	1,50	1,50	1,15	0,95	1,40	1,45	5,00	1,203703704	1
O. smreczynskii Cmol.	UMCS	f	Poland, Lublin	N51.2118	E22.5715	10.06.2021	R. Gosik	3,05	2,50	1,45	1,50	1,05	0,85	1,40	1,50	4,90	1,22	0,966666667
O. smreczynskii Cmol.	UMCS	f	Poland, Lublin	N51.2118	E22.5715	21.07.2021	R. Gosik	2,85	2,35	1,15	1,35	1,00	0,85	1,15	1,65	4,70	1,212765957	0,851851852
O. smreczynskii Cmol.	UMCS	f	Poland, Lublin	N51.2118	E22.5715	21.07.2021	R. Gosik	3,35	2,65	1,50	1,55	1,00	0,90	1,35	1,55	5,10	1,264150943	0,967741935
O. smreczynskii Cmol.	UMCS	f	Poland, Lublin	N51.2118	E22.5715	21.07.2021	R. Gosik	2,85	2,35	1,25	1,35	0,95	0,80	1,15	1,40	4,40	1,212765957	0,925925926
O. smreczynskii Cmol.	UMCS	f	Germany, Berlin	no data	no data	20.08.1968	Z. Cmoluch	3,15	2,70	1,25	1,50	1,35	0,90	1,60	1,65	5,30	1,166666667	0,833333333
O. smreczynskii Cmol.	UMCS	f	Germany, Berlin	no data	no data	20.08.1968	Z. Cmoluch	3,45	2,70	1,60	1,65	1,25	0,95	1,55	1,60	5,40	1,277777778	0,96969697
O. smreczynskii Cmol.	UMCS	f	Germany, Berlin	no data	no data	20.08.1968	Z. Cmoluch	2,90	2,25	1,30	1,30	1,05	0,80	1,55	1,40	4,80	1,288888889	1
O. smreczynskii Cmol.	UMCS	f	Germany, Berlin	no data	no data	20.08.1968	Z. Cmoluch	3,25	2,70	1,50	1,50	1,25	0,90	1,40	1,55	5,10	1,203703704	1
O. smreczynskii Cmol.	CURCI	f	Germany, Hannover-Herrenhausen	N52°23'40''	E9°42'15''	15.05.1989	P. Sprick	3	2,45	1,40	1,50	1,00	0,85	1,40	1,50	4,70	1,224489796	0,933333333
O. smreczynskii Cmol.	CURCI	f	Germany, Hannover, allotment garden area	no data	no data	02.09.1986	P. Sprick	3,2	2,55	1,40	1,50	1,10	0,90	1,40	1,75	5,00	1,254901961	0,933333333
O. smreczynskii Cmol.	CURCI	f	Germany, Hannover-Herrenhausen	N52°23'34''	E9°42'02,7''	19.08.1988	P. Sprick	2,5	2,05	1,10	1,25	0,90	0,75	1,10	1,30	4,00	1,219512195	0,88
O. smreczynskii Cmol.	CURCI	f	Germany, Hannover-Burg/Ledeburg	N52°24'44''	E9°41'56''	26.06.1996	P. Sprick	3,15	2,60	1,40	1,55	1,10	0,95	1,50	1,70	5,00	1,211538462	0,903225806
O. smreczynskii Cmol.	CURCI	f	Germany, Hannover-Herrenhausen	N52°23'40''	E9°42'15''	15.05.1989	P. Sprick	2,9	2,35	1,25	1,40	1,05	0,85	1,30	1,50	4,50	1,234042553	0,892857143
O. smreczynskii Cmol.	CURCI	f	Germany, Hannover-Herrenhausen	N52°23'34''	E9°42'02,7''	19.08.1988	P. Sprick	2,7	2,40	1,30	1,40	1,00	0,85	1,30	1,50	4,40	1,125	0,928571429
O. smreczynskii Cmol.	CURCI	f	Germany, Braunschweig, JKI area	N52°16'34''	E10°33'51''	20.06.2008	P. Sprick	3,05	2,50	1,35	1,45	1,05	0,85	1,30	1,65	4,90	1,22	0,931034483
O. smreczynskii Cmol.	CURCI	f	Germany, Hannover-Herrenhausen	N52°23'40''	E9°42'15''	06.08.1989	P. Sprick	2,85	2,30	1,25	1,40	1,00	0,82	1,30	1,50	4,55	1,239130435	0,892857143
O. smreczynskii Cmol.	CURCI	f	Germany, Hannover-Herrenhausen	N52°23'40''	E9°42'15''	18.05.1989	P. Sprick	3,3	2,60	1,45	1,55	1,10	0,90	1,50	1,50	5,20	1,269230769	0,935483871
O. smreczynskii Cmol.	CURCI	f	Germany, Aerzen-Königsförde	N52°03,58,5'	E9°16'18''	24.08.2021	P. Sprick	3,15	2,55	1,40	1,60	1,10	0,90	1,50	1,80	4,80	1,235294118	0,875
O. smreczynskii Cmol.	CURCI	f	Germany, Hannover-Steintormasch	N52°22'58'	E9°42'31''	12.07.2021	P. Sprick	3,1	2,70	1,45	1,70	1,05	0,95	1,42	1,50	5,00	1,148148148	0,852941176
O. smreczynskii Cmol.	CURCI	f	Germany, Hannover-Steintormasch	N52°22'58''	E9°42'31''	12.07.2021	P. Sprick	3,05	2,60	1,40	1,55	1,10	0,90	1,35	1,75	4,90	1,173076923	0,903225806

O. rotundus Marsh.	UMCS	m	Poland, Lublin	N51.2610	E22.5150	13.07.1971	Z. Cmoluch	2,50	2,00	1,25	1,25	0,95	0,75	1,25	1,35	4,00	1,25	1
O. rotundus Marsh.	UMCS	m	Poland, Lublin	N51.2610	E22.5150	11.06.1973	Z. Cmoluch	2,75	2,20	1,20	1,40	1,00	0,80	1,35	1,50	4,50	1,25	0,857142857
O. rotundus Marsh.	UMCS	m	Poland, Lublin	N51.2610	E22.5150	11.06.1973	Z. Cmoluch	2,45	1,90	1,05	1,20	1,00	0,75	1,20	1,35	4,20	1,289473684	0,875
O. rotundus Marsh.	UMCS	f	Poland, Lublin	N51.2610	E22.5150	17.07.1979	Z. Cmoluch	2,75	2,35	1,25	1,50	0,65	0,75	1,30	1,50	4,40	1,170212766	0,833333333
O. rotundus Marsh.	UMCS	f	Poland, Lublin	N51.2610	E22.5150	17.07.1979	Z. Cmoluch	2,80	2,30	1,35	1,45	1,05	0,90	1,55	1,65	4,40	1,217391304	0,931034483
O. rotundus Marsh.	UMCS	f	Poland, Lublin	N51.2610	E22.5150	17.07.1979	Z. Cmoluch	2,50	2,05	1,25	1,35	0,90	0,75	1,25	1,40	4,30	1,219512195	0,925925926
O. rotundus Marsh.	UMCS	f	Poland, Lublin	N51.2610	E22.5150	17.07.1979	Z. Cmoluch	2,80	2,30	1,45	1,50	1,00	0,90	1,45	1,50	4,20	1,217391304	0,966666667
O. rotundus Marsh.	UMCS	m	Poland, Lublin	N51.2610	E22.5150	17.07.1979	Z. Cmoluch	2,45	2,25	1,10	1,05	0,95	0,75	1,25	1,25	4,20	1,088888889	1,047619048
O. rotundus Marsh.	UMCS	m	Poland, Lublin	N51.2610	E22.5150	17.07.1979	Z. Cmoluch	2,45	2,00	1,25	1,35	1,05	0,80	0,90	1,25	4,30	1,225	0,925925926
O. rotundus Marsh.	UMCS	m	Poland, Lublin	N51.2610	E22.5150	17.07.1979	Z. Cmoluch	2,50	2,20	1,25	1,30	1,05	0,75	1,25	1,20	4,30	1,136363636	0,961538462
O. rotundus Marsh.	UMCS	m	Poland, Lublin	N51.2610	E22.5150	17.07.1979	Z. Cmoluch	2,75	2,25	1,35	1,45	0,85	0,85	1,25	1,40	4,50	1,222222222	0,931034483
O. rotundus Marsh.	UMCS	f	Poland, Lublin	N51.2610	E22.5150	17.07.1979	Z. Cmoluch	3,00	2,50	1,25	1,50	1,00	0,85	1,45	1,50	4,50	1,2	0,833333333
O. rotundus Marsh.	UMCS	f	Poland, Lublin	N51.2610	E22.5150	17.07.1979	Z. Cmoluch	3,10	2,45	1,45	1,45	1,05	0,90	1,45	1,55	4,50	1,265306122	1
O. rotundus Marsh.	UMCS	f	Poland, Lublin	N51.2610	E22.5150	17.07.1979	Z. Cmoluch	2,50	2,20	1,30	1,15	1,05	0,80	1,25	1,35	4,30	1,136363636	1,130434783
O. rotundus Marsh.	UMCS	m	Poland, Lublin	N51.2610	E22.5150	17.07.1979	Z. Cmoluch	2,50	2,15	1,40	1,45	1,20	0,80	1,35	1,40	4,20	1,162790698	0,965517241
O. rotundus Marsh.	UMCS	f	Poland, Milejów	N51.243042	E22.540432	10.06.1983	B. Staniec	3,25	2,40	1,35	1,40	1,10	0,80	1,50	1,50	4,70	1,354166667	0,964285714
O. rotundus Marsh.	UMCS	f	Poland, Milejów	N51.243042	E22.540432	10.06.1983	B. Staniec	2,65	2,15	1,25	1,40	1,20	0,80	1,40	1,50	4,40	1,23255814	0,892857143
O. rotundus Marsh.	UMCS	f	Poland, Gródek	N50.80711	E23.95661	20.07.2005	R. Gosik	2,50	2,05	1,15	1,25	1,10	0,75	1,25	1,20	4,30	1,219512195	0,92
O. rotundus Marsh.	UMCS	f	Poland, Gródek	N50.80711	E23.95661	20.07.2005	R. Gosik	2,55	2,20	1,20	1,25	1,00	0,75	1,00	1,25	4,40	1,159090909	0,96
O. rotundus Marsh.	UMCS	f	Poland, Gródek	N50.80711	E23.95661	20.07.2005	R. Gosik	2,65	2,20	1,25	1,35	1,05	0,80	1,50	1,50	4,40	1,204545455	0,925925926
O. rotundus Marsh.	UMCS	f	Poland, Gródek	N50.80711	E23.95661	09.06.2021	R. Gosik	2,75	2,40	1,25	1,35	1,05	0,85	1,30	1,50	4,40	1,145833333	0,925925926
O. rotundus Marsh.	UMCS	f	Poland, Gródek	N50.80711	E23.95661	09.06.2021	R. Gosik	2,85	2,35	1,15	1,30	1,10	0,85	1,35	1,50	4,50	1,212765957	0,884615385
O. rotundus Marsh.	UMCS	m	Poland, Gródek	N50.80711	E23.95661	09.06.2021	R. Gosik	2,30	2,00	1,15	1,25	0,85	0,65	1,25	1,50	4,20	1,15	0,92
O. rotundus Marsh.	UMCS	f	Poland, Gródek	N50.80711	E23.95661	09.06.2021	R. Gosik	3,00	2,45	1,25	1,50	1,00	0,80	1,35	1,40	4,50	1,224489796	0,833333333
O. rotundus Marsh.	UMCS	f	Poland, Gródek	N50.80711	E23.95661	09.06.2021	R. Gosik	3,00	2,55	1,20	1,50	1,00	0,80	1,30	1,35	4,50	1,176470588	0,8
O. rotundus Marsh.	UMCS	f	Poland, Gródek	N50.80711	E23.95661	09.06.2021	R. Gosik	3,25	2,50	1,15	1,50	1,10	0,85	1,30	1,50	4,70	1,3	0,766666667
O. rotundus Marsh.	UMCS	m	Poland, Gródek	N50.80711	E23.95661	09.06.2021	R. Gosik	2,40	2,00	1,10	1,30	1,00	0,65	1,25	1,35	4,10	1,2	0,846153846
O. rotundus Marsh.	UMCS	f	Poland, Gródek	N50.80711	E23.95661	09.06.2021	R. Gosik	2,80	2,30	1,25	1,35	1,05	0,85	1,20	1,50	4,20	1,217391304	0,925925926
O. rotundus Marsh.	UMCS	f	Poland, Gródek	N50.80711	E23.95661	09.06.2021	R. Gosik	2,85	2,40	1,25	1,50	1,05	0,70	1,25	1,50	4,30	1,1875	0,833333333

O. rotundus Marsh.	UMCS	f	Poland, Gródek	N50.80711	E23.95661	09.06.2021	R. Gosik	2,30	2,00	1,30	1,40	1,10	0,80	1,25	1,55	4,10	1,15	0,928571429
